# On-Chip Tunable Cell Rotation Using Acoustically Oscillating Asymmetrical Microstructures

**DOI:** 10.3390/mi9110596

**Published:** 2018-11-14

**Authors:** Lin Feng, Bin Song, Deyuan Zhang, Yonggang Jiang, Fumihito Arai

**Affiliations:** 1 School of Mechanical Engineering & Automation, Beihang University, Beijing 100191, China; linfeng@buaa.edu.cn (L.F.); songb@buaa.edu.cn (B.S.); zhangdy@buaa.edu.cn (D.Z.); 2 Beijing Advanced Innovation Center for Biomedical Engineering, Beihang University, Beijing 100083, China; arai@mech.nagoya-u.ac.jp; 3 Department of Micro-Nano Mechanical Science & Engineering, Nagoya University, Nagoya 464-0814, Japan

**Keywords:** micromachine, cell rotation, acoustic waves, acoustic streaming, on-chip manipulation

## Abstract

The precise rotational manipulation of cells and other micrometer-sized biological samples is critical to many applications in biology, medicine, and agriculture. We describe an acoustic-based, on-chip manipulation method that can achieve tunable cell rotation. In an acoustic field formed by the vibration of a piezoelectric transducer, acoustic streaming was generated using a specially designed, oscillating asymmetrical sidewall shape. We also studied the nature of acoustic streaming generation by numerical simulations, and our simulation results matched well with the experimental results. Trapping and rotation of diatom cells and swine oocytes were coupled using oscillating asymmetrical microstructures with different vibration modes. Finally, we investigated the relationship between the driving voltage and the speed of cell rotation, showing that the rotational rate achieved could be as large as approximately 1800 rpm. Using our device, the rotation rate can be effectively tuned on demand for single-cell studies. Our acoustofluidic cell rotation approach is simple, compact, non-contact, and biocompatible, permitting rotation irrespective of the optical, magnetic, or electrical properties of the specimen under investigation.

## 1. Introduction

Cell manipulations have attracted great attention in the field of modern bioscience [1,2] because the cell is the basic structural, functional, and biological unit of all known living organisms. Cell rotation is one of the fundamental techniques of cell manipulations, which plays a key role in various fields, including cell observation [3], cell analysis [4,5,6], and drug discovery [7]. For example, diatom cells can be set in an ordered alignment by specific rotational manipulation, which is utilized to achieve detection in screening, diagnosis, and medicine. In addition, since spindle observation is very important during meiosis to study its dynamic behavior and obtain other information, it should be oriented to a specific angle via cell rotation for observation under a polarization microscope [8]. Furthermore, controllable cell rotation is also required for the removal or the transfer of nuclei in transgenic animal research [9,10]. For instance, after enucleation by chemical treatment, an observation via high-speed cell rotation must be performed to confirm whether the nuclei of cells have been removed successfully, because the nucleus of an oocyte is anisotropically located at the cell edge, which is not visible in typical translational manipulations. Thus, cell rotation has become a powerful tool for cell observation.

Different approaches have been adopted to achieve rotational manipulation of microparticles and cells. Conventionally, two micromanipulators are used to perform motions. Under a microscope, one micromanipulator immobilizes the target cell, while the other applies a torque for the rotation [11,12]. However, this operation requires expert operators, which reduces its applicability and repeatability. Lin Feng et al. succeeded in cell rotation using dual-arm magnetically driven microtools, but this technique requires a complex control system [13]. Although optical tweezers have been successfully used to rotate cells, damage to the biological samples by laser-induced heating may be observed, and a sufficiently strong torque to rotate larger cells (>20 μm) may not be generated [14,15]. In the electrorotation technique, an electric field is applied to induce a torque to the target cell by patterning multiple electrodes [16,17,18]. Nevertheless, these custom-designed electrode structures are rather complex to fabricate, and this approach may destroy biological species by current-induced heating. However, as of yet, no detailed research has been performed on high-speed rotational manipulation of big-size cells like swine oocytes.

Acoustic waves have recently offered a promising alternative technique for force generation at the microscale level and have been widely employed for several applications [19,20,21]. Acoustofluidic devices that fuse acoustics and microfluidics have the potential to dramatically improve methods for manipulating cells and small biological samples. For example, an oscillating solid structure placed in a microfluidic environment generates a local acoustic flow [22,23,24]. Ozcelik et al. used vibrating sharp-edged microstructures to engender two counter-rotating microvortices in the surrounding fluid [25]. However, this acoustofluidic device merely achieved the rotational manipulation of HeLa cell (≈20 μm) using oscillating solid structures and was unable to perform the rotation of big-size cells like oocytes (>80 μm). Hayakawa et al. adopted micropillar patterns to accomplish the rotation of mouse oocytes based on a vibration-induced whirling flow [26,27]. Nonetheless, the target cells were not retrieved from the microchip, and the flexibility in adjusting the rotational speed was hard to control. Ahmed et al. trapped air microbubbles within predefined sidewall microcavities inside a microchannel utilized to rotate cells. A drawback of the microbubbles is their enlargement over time, which eventually shifts their resonance frequency [28,29].

Thus, we, herein, aim to create adequately robust, stable, and controllable forces to manipulate the high-speed rotation of big-size cells (≈100 μm) by designing custom-made structures. We propose an on-chip cell rotation technology using acoustic waves, which integrates the rotation mechanisms of ultrasonic vibration into a microfluidic chip. Compared with other technologies, this method is robust, biocompatible, easy to control, and independent of the optical, magnetic, or electrical properties of the sample. It allows an effective and precise rotation of specimens over a wide range of sizes, shapes, and properties.

**Figure 1:**
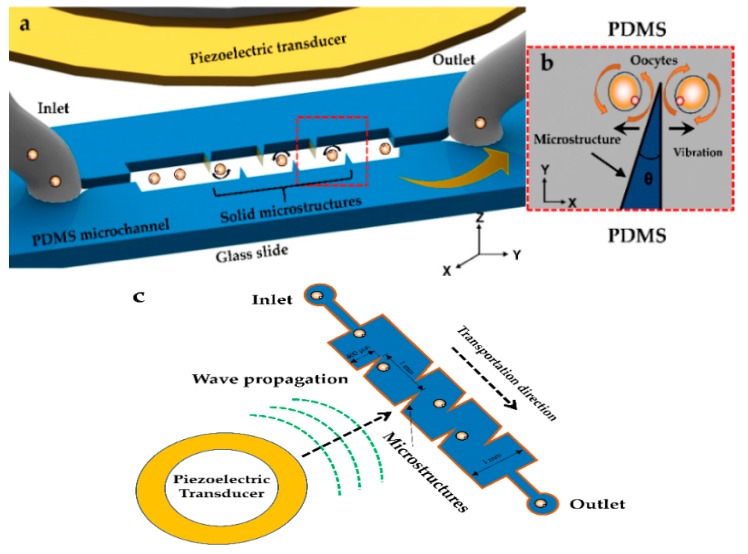
Design of the rotational device using acoustic waves: (**a**) conceptual overview of the microfluidic chip and (**b**) oscillations of the solid microstructures used for cell rotation; (**c**) working principle and typical geometric dimensions of the acoustofluidic rotational manipulation device.

Figure 1a shows the design of the microfluidic chip driven by acoustic flows, including a polydimethylsiloxane (PDMS) microchannel with six microstructures and a piezoelectric transducer that provides the acoustic energy. Inlet and outlet holes were punched into the PDMS chip and used to load and unload cells. The length, width, and depth of the microchannel were 5 mm, 1 mm, and 200 μm, respectively (Figure 1c). Considering the diameter of our experimental samples and previous researches performed by Adem Ozcelik’s group [25], each microstructure was designed to be of a constant length of 400 μm, a tip angle (θ) of 20°, and a depth of 200 μm, which was used to generate acoustic streaming for cell rotation (Figure 1b). The piezoelectric transducer and the microfluidic chip were bonded on a glass slide (150 μm). Our experimental objects were diatom cells (≈80 μm) and swine oocytes (≈100 μm). Figure 1c also illustrates that, when acoustic waves are excited by a piezoelectric transducer, vibration-induced local acoustic flows are generated in the surrounding fluid because of viscous dissipation in the microchannel. Moreover, the response to time-harmonic forcing is generally not harmonic because of the dissipative property of the fluid. The fluid’s response to harmonic forcing can be viewed as a combination of a time-harmonic response, generally referred to as acoustic response, and the remainder, referred to as acoustic streaming. The latter is a byproduct of the acoustic attenuation caused by viscous dissipation; hence, it provides a unique method of utilizing the dominant viscous nature of microfluidic flows [30,31,32,33]. With the growing use of cell-on-chip tools for investigating microparticles and cells, our method is anticipated to be an invaluable tool in biology, biophysics, and medicine.

## 2. Materials and Methods

### 2.1. Theoretical Analysis

Numerical simulations were performed to theoretically analyze the mechanisms and properties of acoustic streaming generation [32,33]. Vectors and scalars are represented by bold and regular fonts, respectively. First, the continuity and momentum equations are as follows:

(1) $$\frac{\partial \rho }{\partial t}+\nabla \left(\rho v\right)=0$$

(2) $$\rho \left[\frac{\partial v}{\partial t}+\left(v\cdot \nabla \right)v\right]+v\nabla \cdot \left(\rho v\right)=-\nabla p+\left(\zeta +\frac{4}{3}\eta \right)\cdot v-\eta \nabla \times \nabla \times v$$

where *ρ* is the mass density, ***v*** is the velocity of fluid, *p* is the pressure of fluid, and ζ and η are bulk and shear dynamic viscosities, respectively. The relation between *ρ* and *p* was assumed to be linear:

(3) $$p=c_{0}^{2}\rho$$

where *c*_0_ is the velocity of sound in the fluid at rest. We employed Nyborg’s perturbation technique [34] and expanded the field of fluid velocity, density, and pressure as follows:

(4a) $$v=v_{0}+v_{1}+v_{2}+\cdots \cdots$$

(4b) $$\rho =\rho_{0}+\rho_{1}+\rho_{2}+\cdots \cdots$$

(4c) $$p=p_{0}+p_{1}+p_{2}+\cdots \cdots$$

where *ρ*_0_ is the density of the fluid at rest and a constant, ***v*_1_** is the first-order sound velocity, and *ρ*_2_ and ***v*_2_** are the second-order parameters. Substituting Equations (3) and (4) into Equations (1) and (2), respectively, the following equations, referred to as the first- and second-order equations of acoustics, are obtained:

(5) $$\frac{\partial \rho_{1}}{\partial t}+\rho_{0}\left(\nabla v_{1}\right)=0$$

(6) $$\rho_{0}\frac{\partial v_{1}}{\partial t}=-\nabla p_{1}+\left(\zeta +\frac{4}{3}\eta \right)\cdot v_{1}-\eta \nabla \times \nabla \times v_{1}$$

and

(7) $$\langle \frac{\partial \rho_{2}}{\partial t}\rangle +\rho_{0}\nabla \cdot \langle v_{2}\rangle +\nabla \cdot \langle \rho_{1}v_{1}\rangle =0$$

(8) $$\begin{array}{r} \rho_{0}\langle \frac{\partial v_{2}}{\partial t}\rangle +\langle \frac{\partial }{\partial t}\left(\rho_{1}v_{1}\right)\rangle +\rho_{0}\langle v_{1}\cdot \nabla v_{1}\rangle +\rho_{0}v_{1}\nabla \cdot v_{1}\\ =-\nabla \langle p_{2}\rangle +\left(\zeta +\frac{4}{3}\eta \right)\cdot \langle v_{2}\rangle -\eta \nabla \times \nabla \times \langle v_{2}\rangle \end{array}$$

where 〈x〉 denotes the time average of quantity x over a full oscillation time period. As pointed out in Equations (5)–(8), the time averages of the continuity and momentum equations were not zero, indicating the streaming velocity of the fluid. Moreover, note that these equations, which were complemented with appropriate boundary conditions, were numerically solved using the commercial finite element method ANSYS FLUENT 17.0 (Canonsburg, PA, USA) to characterize the acoustic streaming distributions in the vicinity of the microstructures. Further details would be discussed in the subsequent part. Consequently, the real mechanism of formation of acoustic streaming is caused by viscous dissipation and non-linear absorption in the microchannel.

### 2.2. Simulation and Observed Trajectories

**Figure 2:**
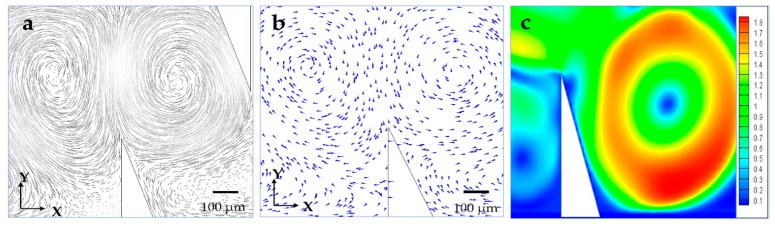
Simulation results of the velocity vector field in the microfluidic chip using FLUENT: the arrow represents the direction of motion of (**a**) the acoustic microstreaming around the oscillating microstructure and (**b**) the flow direction of microstreaming from the X–Y plane of the oscillating microstructure; (**c**) simulation results of the velocity fields, unit: m/s.

**Figure 3:**
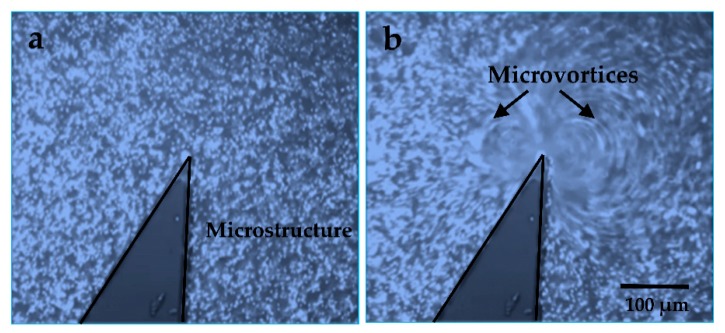
Experimentally observed trajectories of the 1 μm diameter fluorescent beads in our acoustically vibrated microstructure: (**a**) the driving voltage is 0 V_p-p_, while in (**b**), the driving voltage is 20 V_p-p_.

Finite element method (FEM) simulations for modeling the local microstreaming in the presence of microstructures are used to confirm this unique phenomenon under the standing acoustic wave. Figure 2a–c show the simulation results of the oscillating solid microstructure at 4.6 kHz using ANSYS FLUENT 17.0, (type of mesh used: all triangles method, mesh size: 10 μm, boundary conditions: outflow). With this method, two counter-rotating microvortices were generated by setting up the oscillation function in the X–Y plane, which matched well with our previous numerical analysis. Accordingly, 1 μm diameter fluorescent polystyrene spherical beads (PSRF01000, ZHONGKELEIMING Co., Ltd., Beijing, China) were subjected to the experimental system to confirm the fluid field distribution around the solid microstructures (10 µL of the particle suspension was centrifuged and washed with deionized water three times. Then, the microsphere suspensions were diluted to 100 µL using deionized water). Figure 3a,b show that a vibrating solid structure induced two local acoustic flows in its vicinity when a sine wave was applied, and the driven oscillation was harmonic with a frequency equal to 4.6 kHz (Video S1, Supplementary Materials). Moreover, the size of the microvortices on both sides of the solid microstructure was different because of the asymmetric design, which was utilized to perform different manipulations in a low-power acoustic field in the following experiments.

## 3. Experiments and Results

### 3.1. System Setup

**Figure 4:**
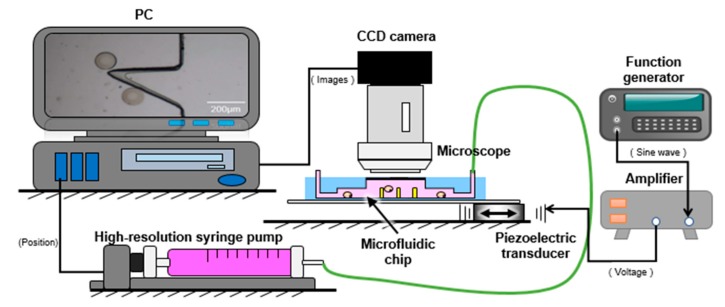
Components of the experimental system.

Figure 4 shows an overview of the system components of the manipulation platform, consisting of the observation and driving systems. A charge-coupled device (CCD) camera (GS3-U3-23S6C-C, Point Grey Co., Ltd., Richmond, BC, Canada) was attached to a differential interference contrast (DIC) microscope (CX41, Olympus Co., Ltd., Tokyo, Japan) to observe the manipulation better. A control personal computer was used to acquire real-time and stereoscopic images. The microfluidic chip was fixed on a stage. The other system included three parts. A function generator (Wave Station 2012, LECROY, New York, NY, USA) sent a sine wave to a high-voltage amplifier (ATA-2042, Agitek, Xi’an, China). This output signal was then used to drive the piezoelectric transducer. Meanwhile, a high-resolution syringe pump (PHD ULTRA NANOMITE, Harvard, Cambridge, MA, USA) was applied to the inlet of the PDMS channel, which could inject micro and quantitative liquid to transport cells steadily and accurately.

**Figure 5:**
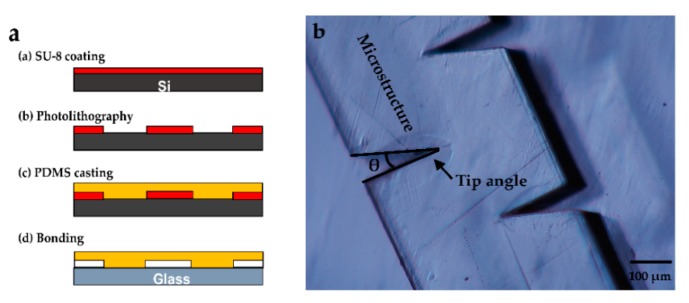
Microfluidic chip fabrication: (**a**) fabrication process of the microfluidic chip and (**b**) digital microscopic image of the solid microstructure mold (θ = 20°).

Figure 5a shows the fabrication process of the microfluidic chip. First, the photoresist SU-8 (GM1075, Resemi, Suzhou, China) was coated on the substrate at 1000 rpm for 90 s. The SU-8 mold was developed after exposure (see Figure 5b). A Sylgard 184 silicone elastomer base was mixed with Sylgard 184 silicone elastomer curing agent (Dow Corning, Midland, MI, USA) at a ratio of 10:1 and cured at 70 °C for 2 h to form the PDMS channels. Subsequently, the surface of the PDMS channels and a 40 mm × 50 mm × 0.15 mm (width × length × thickness) glass slide were treated with oxygen plasma for 20 s and bonded at 90 °C for 10 min. Finally, a piezoelectric transducer (81-7BB-27-4L0, Murata Electronics, Kyoto, Japan) was attached on the back of the glass and adjacent to the PDMS channels using epoxy (84101, Permatex, Hartford, CT, USA).

### 3.2. Rotational Manipulation

**Figure 6:**
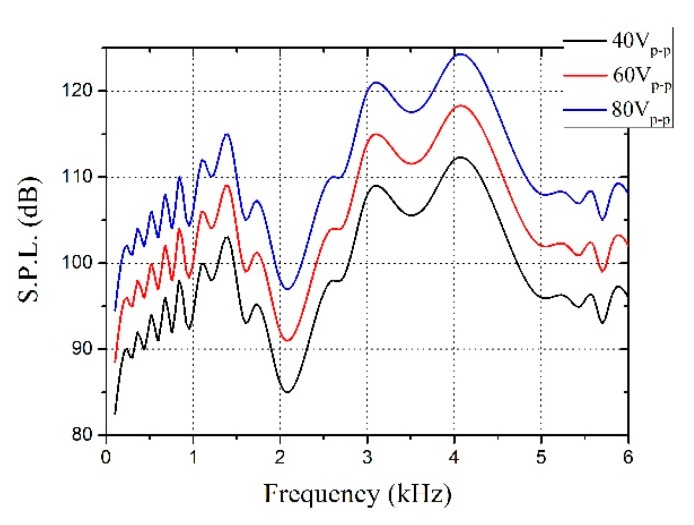
Relationship between sound pressure level (S.P.L.) and acoustic frequency (distance from the sound source: 10 cm).

In Figure 6, the sound pressure level (S.P.L.) was different because the vibration amplitude of the piezoelectric transducer varied with changes of the driving frequency, which illustrated that the S.P.L. was closely related to acoustic energy. The piezoelectric transducer was driven by applying the voltage; hence, the S.P.L. increased upon increasing the voltage within limits (Figure 6). Therefore, 20 V_p-p_ sine wave voltage was applied on the piezo, and the frequency in high S.P.L. was selected to conduct experiments on evident experimental phenomena.

**Figure 7:**
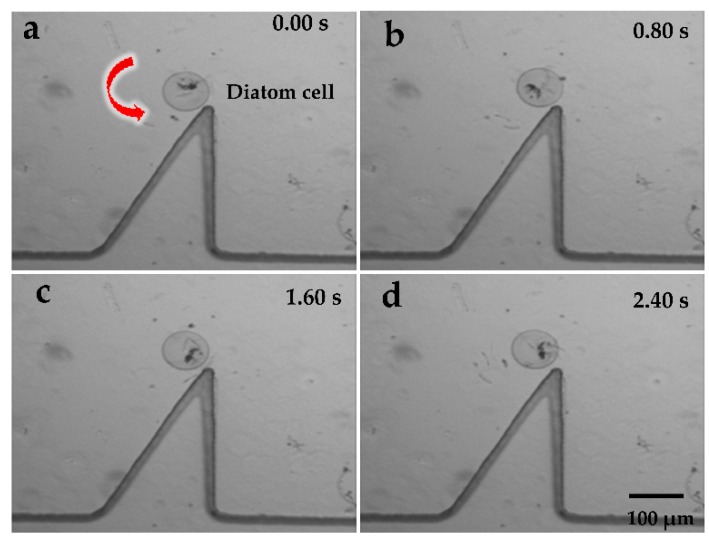
(**a**–**d**) Rotational manipulation of the diatom cell at 4.6 kHz and 20 V_p-p_.

**Figure 8:**
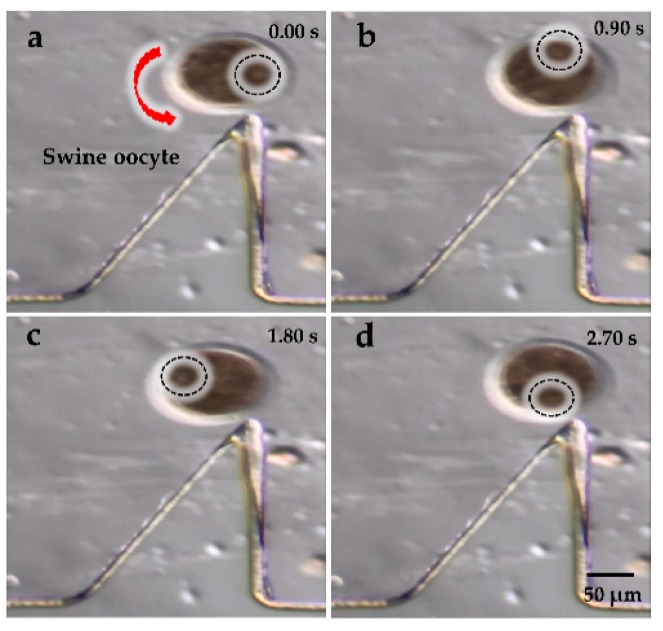
(**a**–**d**) Rotational manipulation of the swine oocyte at 4.6 kHz and 20 V_p-p_.

Figure 7a–d and Figure 8a–d show the rotational manipulation of the diatom cell and the swine oocyte, respectively (Video S1, Supplementary Materials). At the tip of the oscillating solid microstructure, a strong torque was created at 4.6 kHz and 20 V_p-p_ (peak-to-peak voltage value), which was used to achieve cell rotation. Considering different sizes of the swine oocyte and the diatom cell, the rotational speed is well-known to decrease upon increasing the cell diameter in the same driving voltage. In addition, when the applied frequency was changed to 4.2 kHz, the microstreaming orientation was switched to a clockwise direction (Video S1, Supplementary Materials). Qiang Tang et al. investigated patterns of the acoustic streaming field at different frequencies and proved that, as the frequency increased, the vibrating pattern became more complex, which could result in different orientations of the acoustic streaming field [35]. Such behavior is attributed to the change of the vibrating pattern. At different applied frequencies, the oscillating microstructure provides different types of acoustic wave patterns because the acoustic streaming structure is strongly influenced by the vibration source which provided acoustic energy. Consequently, the location of the exciting source and corresponding boundaries will change according to previous researches [36,37], leading to localized reversed direction of the acoustic streaming vortex.

**Figure 9:**
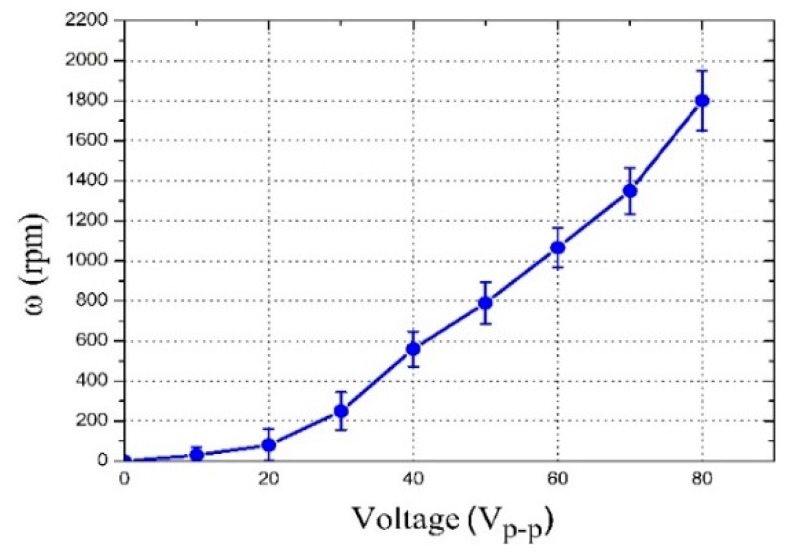
Relationship between the rotation speed of the diatom cells and the voltage, showing that the former can be tuned by the applied peak-to-peak voltage.

We also examined the relationship between the diatom cell rotational speed and the driving voltage. The voltage was adjusted by 10 V_p-p_ every time from 20 to 80 V_p-p_, using an amplifier at 4.6 kHz. The tip angle, channel width, and depth were experimentally optimized to 20°, 1000, and 200 μm, respectively. Figure 9 illustrates that the rotational speed of the diatom cells increased with the increasing voltage, which could be as large as, approximately, 1800 rpm. The flexibility of the rotational speed supports its wide utilization in single-cell studies, where the shear force for the rotation can be tuned on-demand. In a micro/nanometer-size world, the viscous force and other surface forces exert a dominant impact on cell manipulation, while the volume and inertia forces hardly have any effect. Therefore, the cells rarely maintain the rotation when the supply is cut off because of the strong viscosity, which could easily control the specific cell position and orientation (Video S1, Supplementary Materials).

Finally, we needed to assess the effect of the proposed method driven by acoustic waves on the viability of cells. On the basis of previous experiments by our group, we transported and rotated five oocytes with our combined system for five minutes. The target oocytes were collected and incubated in a culture well at 37 °C in a 5% CO_2_ atmosphere for three hours. The viability of the incubated cells was then assessed using the LIVE/DEAD Viability Kit (L-3224, Life Technologies Japan Ltd., Tokyo, Japan). All experimental samples remained viable after the incubation. Thus, the proposed method did not affect cell viability [26].

### 3.3. Cell Trapping

**Figure 10:**
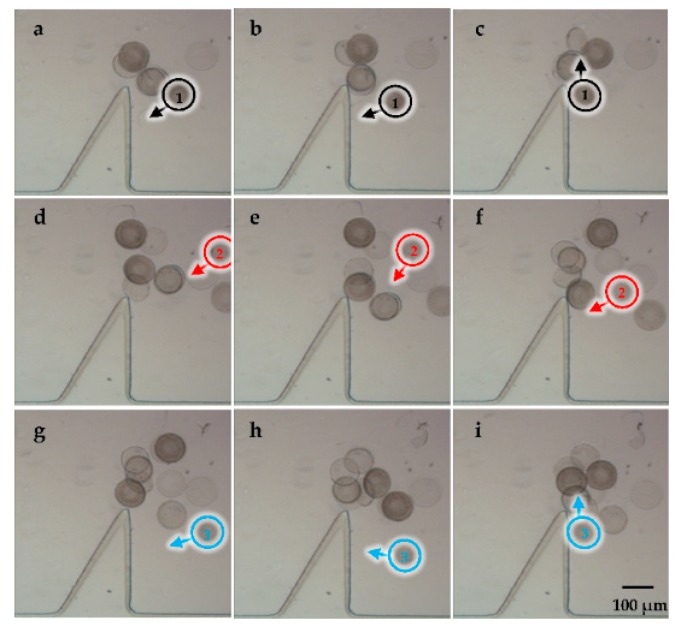
(**a**–**i**) Microscopic images of the multiple diatom cells trapped by adjusting the frequency.

**Figure 11:**
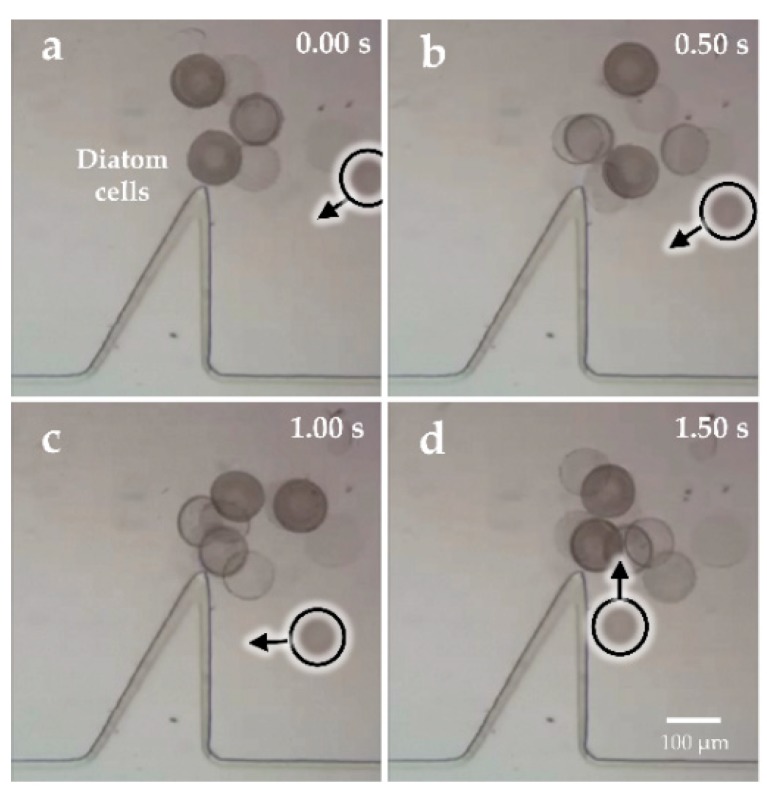
(**a**–**d**) Microscopic images showing the time necessary to trap a diatom at the distance of 400 µm.

Figure 10 shows cell trapping during the transportation by adjusting different frequencies, which effectively settled the problem about transporting the cell to a certain position with high precision (Video S1, Supplementary Materials). The cell was trapped in a special trajectory driven by acoustic flows when we applied a vibration with the frequency of 4 kHz at the voltage of 40 V_p-p_. Especially, the right-side structure was able to form stronger acoustic streaming than the other side, which was utilized to trap cells in a lower voltage. Figure 11 shows that the time required for successfully trapping a cell at a distance of 400 μm was only approximately 1.5 s and would continue to reduce with the increasing driving voltage.

## 4. Conclusions

This study describes a rotational manipulation method using acoustic waves in a microfluidic chip. We also successfully demonstrated the feasibility of non-invasive and non-contaminated trapping and rotation of diatom cells and swine oocytes at different vibration modes. The torque created by a specially designed oscillating microchannel shape with triangles was controlled by adjusting the voltage applied to the piezoelectric transducer, which can be used for various applications by choosing the required rotational speeds. Our future work will be focused on achieving a three-dimension rotation of oocytes with increased precision and motion stability.

## Supplementary Materials

The following are available online at http://www.mdpi.com/2072-666X/9/11/596/s1, Video S1: Cell manipulation.
